# Review of Current and Future Medical Treatments in Head and Neck Squamous Cell Carcinoma

**DOI:** 10.3390/cancers16203488

**Published:** 2024-10-15

**Authors:** Aaron M. Lee, Alice N. Weaver, Phillip Acosta, Lauren Harris, Daniel W. Bowles

**Affiliations:** 1 Division of Medical Oncology, University of Colorado School of Medicine, Aurora, CO 80045, USA; aaron.m.lee@cuanschutz.edu (A.M.L.); alice.weaver@cuaschutz.edu (A.N.W.); 2 Department of Medicine, University of Colorado School of Medicine, Aurora, CO 80045, USA; phillip.acosta@cuanschutz.edu; 3 University of Colorado School of Medicine, Aurora, CO 80045, USA; lauren.a.harris@cuanschutz.edu; 4 Rocky Mountain Regional Veterans Affairs Medical Center, Aurora, CO 80045, USA

**Keywords:** head and neck squamous cell carcinoma, immunotherapy, targeted therapy

## Abstract

**Simple Summary:**

The landscape for medical therapy in head and neck squamous cell carcinoma has considerably evolved in the last few decades. While early-stage disease remains primarily managed with surgery and/or radiation therapy, immunotherapy and antibody-based therapy have been transformative and practice-changing for patients with recurrent and/or metastatic disease. Unfortunately, many patients ultimately develop resistance to these approaches, with limited options for subsequent treatment. A wide breadth of novel therapies has shown promise in preclinical and early-phase clinical settings, many of which are now undergoing translation into the clinical arena. Here, we provide a review of how medical therapy is used in the management of HNSCC, with a specific focus on active clinical trials, to inform the medical oncologist treating patients with this disease.

**Abstract:**

Head and neck squamous cell carcinoma (HNSCC) is a complex cancer requiring a multidisciplinary approach. For patients with locally or regionally advanced disease, surgery and/or radiation are the cornerstones of definitive treatment. Medical therapy plays an important adjunct role in this setting, typically consisting of a platinum-based regimen given as induction, concurrent, or adjuvant treatment. While relapsed/metastatic HNSCC has historically been a difficult-to-treat disease with poor outcomes, options have considerably improved with the incorporation of biologics and immune checkpoint inhibitors. Clinical trials are ongoing to investigate novel approaches, including new and combination immunotherapies, targeted therapies, therapeutic vaccines, antibody–drug conjugates, and cellular therapies. The results thus far have been mixed, highlighting the knowledge gaps that continue to challenge the medical oncologist treating HNSCC. Here, we present the most updated and broad review of the current treatment landscape in both locoregional and metastatic HNSCC and discuss the expansive future medical therapies under investigation.

## 1. Introduction

Head and neck squamous cell carcinoma (HNSCC) is a common, yet difficult to manage, cancer. Head and neck cancers are cancers that arise from mucosal epithelial cells of the oral cavity, oropharynx, pharynx, and hypopharynx. About 54,000 new cases of HNSCC were diagnosed in the United States in 2022, and HNSCC ranks as the seventh most prevalent cancer globally [1]. Common risk factors for the development of HNSCC include inhaled and smokeless tobacco, excessive alcohol use, and viral infections, particularly human papillomavirus (HPV), a common cause of oropharyngeal HNSCC. The incidence of HNSCC is increasing in the developing world, largely attributable to tobacco and alcohol use, while HNSCC in developed countries is more often HPV-related [1]. While early-stage disease may be managed with surgical resection or radiation therapy (RT) alone, it is common for patients to present with locally advanced disease requiring a multidisciplinary approach. In such cases, referral to a high-volume and/or tertiary care center should be strongly considered; in fact, treatment at an academic comprehensive cancer program is associated with improvements in survival outcomes [2]. Here, we review the medical management of locoregional and metastatic HNSCC, including chemotherapy and biologic therapy, as well as emerging areas of interest such as novel immunotherapy, targeted therapies, therapeutic vaccines, and cellular therapy.

## 2. Overview of Medical Treatment in Locoregional Disease

Locoregional HNSCC requires multimodality therapy. Initial treatment decisions revolve around whether upfront surgery is possible and indicated. This, in turn, depends on the site of origin and extent of disease. For example, surgical resection is the preferred approach for almost all HNSCCs of the oral cavity. In tumors arising from the oropharynx, surgical resection may be preferred for earlier stage disease, while definitive chemoradiation is favored for larger primary tumors and/or disease with more significant neck involvement [3]. Either surgery or definitive chemoradiation can be considered for cancers of the hypopharynx, depending on whether laryngeal organ preservation is possible. Patients treated with surgery may require adjuvant therapy consisting of radiation with or without chemotherapy, depending upon the stage and pathologic findings after resection. Similarly, patients treated with definitive chemoradiation with an incomplete response may require salvage resection [3].

### 2.1. Cisplatin with Concurrent RT

When concurrent systemic therapy with radiation is indicated for locoregionally advanced HNSCC, cisplatin is the preferred agent. Cisplatin plus radiation, either as definitive or post-operative therapy, improves overall survival (OS) compared with radiation alone, as proven in multiple studies (Table 1) [4,5,6]. This standard of care was established decades ago when chemotherapy choices were more limited and, as such, there are few studies comparing cisplatin head-to-head with alternative agents. Although cisplatin is the clear choice for systemic therapy plus RT in locoregional disease, there is some disagreement as to the best schedule of administration. Two common regimens are cisplatin 100 mg/m^2^ given once every three weeks for three doses, also known as “high-dose” or “bolus-dose” cisplatin, and cisplatin 30–40 mg/m^2^ given weekly for up to seven doses. Weekly cisplatin is hypothesized to be more tolerable, but there are fewer data supporting the efficacy of this schedule. A study from the Japanese Head and Neck Cancer Study Group showed the non-inferiority of weekly cisplatin compared with every-three-week cisplatin with respect to overall survival, along with a lower frequency of grade 3 side effects [7]. Multiple other studies have failed to replicate these results, showing inferior outcomes from weekly cisplatin despite improvements in tolerability [8,9]. At present, cisplatin given every three weeks is the gold standard for concurrent chemoradiation. Weekly cisplatin can be considered on a case-by-case basis in patients who may not tolerate bolus-dosing, with the goal of achieving a cumulative cisplatin dose of at least 200 mg/m^2^ [10]. Additional trials are ongoing to better understand the efficacy and tolerability of weekly versus every-three-week cisplatin administration.

### 2.2. Options for Patients Ineligible for Cisplatin

For patients who are unable to receive cisplatin due to functional status and/or co-morbidities, there are multiple alternative options for chemoradiation. However, studies comparing these various regimens are lacking. Carboplatin-based combination therapy with either 5-Fluorouracil (5-FU) or paclitaxel has activity in the definitive chemoradiation setting and is an option for patients who cannot receive cisplatin but have good functional status otherwise [11,15,16]. A recent study also showed a benefit for docetaxel when added to RT compared with RT alone in cisplatin-ineligible patients [17]. There are some retrospective studies demonstrating efficacy for single-agent carboplatin given with RT in this setting, but clinical use remains off-label due to a lack of strong prospective data.

If chemotherapy is generally not deemed to be a good option, the epidermal growth factor receptor (EGFR) inhibitor cetuximab is another alternative. Cetuximab plus radiation resulted in an overall survival of 49 months compared with 29.3 months with RT alone (HR 0.73) in a pivotal trial leading to its approval [18]. These results generated strong interest in using cetuximab as a less-toxic alternative or adjunct to chemotherapy, but subsequent studies did not support cetuximab in routine clinical management. Cetuximab added to cisplatin and RT increased toxicity without significantly improving survival in a study of locally advanced HNSCC, and similar results were observed when cetuximab was added to carboplatin plus 5-FU and RT [19,20]. In addition, a de-intensification approach of cetuximab plus RT failed to show non-inferiority when compared with cisplatin plus RT in patients with HPV-positive HNSCC and had similar overall rates of toxicity [21,22,23]. Nevertheless, cetuximab plus RT remains an option for selected patients undergoing treatment with systemic therapy plus concurrent RT for HPV-negative cancers when cytotoxic chemotherapeutic agents cannot be used. Cetuximab is not recommended for HPV-positive HNSCC (Table 1).

### 2.3. Options and Indications for Induction Therapy

Induction chemotherapy followed by RT or surgery can be considered in HNSCC although there is a considerable debate regarding best practices for use. The potential benefits of induction chemotherapy include a reduced rate of distant metastases and decreased surgical extent for patients who have a favorable response [24]. The latter is of particular importance for patients for whom a total laryngectomy would otherwise be recommended, and induction chemotherapy as part of a larynx-preservation strategy in selected patients with hypopharyngeal and laryngeal cancers is listed as a category 2A recommendation in the NCCN guidelines [3]. However, the OS benefit has not been demonstrated for induction chemotherapy, so these benefits must be weighed against the possibility of significant treatment-related toxicity and an extension of the overall treatment time. When induction chemotherapy is used, the triplet regimen of cisplatin, 5-FU, and a taxane (usually docetaxel) is preferred, based on multiple randomized controlled trials showing improvements in response rates (RR), disease-free survival (DFS), and OS when compared with the traditional doublet of cisplatin plus 5-FU [25]. An alternative triplet of cetuximab, carboplatin, and paclitaxel can also be considered for HPV-negative disease. It is unclear whether, and which, systemic therapy should be given concurrently with definitive RT following induction chemotherapy.

### 2.4. Immunotherapy in the Locoregional Disease Setting

Immune checkpoint inhibition (ICI) has been transformative for many cancers [26], but early studies in locally advanced HNSCC showed mixed results. For patients undergoing definitive chemoradiation, the addition of the anti-PD-L1 monoclonal antibodies pembrolizumab or avelumab did not improve progression-free survival (PFS) or OS in a series of trials [27,28,29]. In contrast, immunotherapy in the neoadjuvant setting may provide clinical benefits. An HNSCC cohort of CheckMate 358 noted pathologic responses following neoadjuvant nivolumab, an anti-PD-1 monoclonal antibody [30], and patients with high-risk resectable HNSCC receiving neoadjuvant pembrolizumab who achieved a pathologic response had lower rates of recurrence [31,32]. The CIAO trial also reported major pathologic responses following neoadjuvant durvalumab (anti-PD-L1) with or without tremelimumab (anti-CTLA-4) in patients with HNSCC [33,34]. In summary, neoadjuvant immunotherapy appears to have potential utility, albeit with potentially increased adverse effects [35]. A long-term follow-up is needed to better understand the impact of pathologic responses on adjuvant therapy recommendations and the risk of recurrent/metastatic disease. There are limited data to support the use of purely adjuvant immunotherapy. The phase III IMvoke010 (NCT03452137) study of adjuvant atezolizumab, an anti-PD-1 monoclonal antibody, was terminated due to the failure to meet the primary endpoint of PFS [36]. ADJORL1 is a phase II trial evaluating nivolumab with or without ipilimumab (anti-CTLA-4) following salvage surgery in HNSCC and is actively recruiting (NCT03406247). Thus, while interest in immunotherapy for locoregional HNSCC remains high, this approach is still under investigation to determine the optimal timing of immunotherapy exposure relative to other treatment modalities. It remains unclear why immunotherapy has not demonstrated more clinical utility in the locoregional setting although one hypothesis is that radiation and chemotherapy may have local immunosuppressive effects, thereby dampening the effect of immunotherapy in localized disease.

### 2.5. Circulating Tumor Tissue HPV DNA as a Predictive Biomarker in HNSCC

Surveillance for HNSCC is critical as 15–25% of patients with HPV-related HNSCC develop recurrent disease following the initial treatment. A multimodality approach of a physical exam and imaging studies are necessary for surveillance. Cell-free tumor DNA has garnered considerable interest in many malignancies as a means for the non-invasive detection of cancer, but has not yet found a role in HNSCC. There has been a growing interest in the utility of circulating tumor tissue modified viral (TTMV)-HPV DNA as a biomarker for use as a diagnostic or surveillance tool. Currently, NavDx (Naveris) is the only clinically validated TTMV-HPV DNA blood test available. One retrospective case series of 1076 patients with previously treated oropharyngeal SCC (OPSCC) noted a positive predictive value (PPV) of 95.0%; notably, TTMV-HPV DNA was the first indicator of recurrence for 72% of patients [37]. A separate retrospective observational cohort study evaluating TTMV-HPV DNA in OPSCC surveillance observed negative predictive values (NPVs) of 99.4% per text and 98.4% per patient [38]. As a diagnostic tool, one retrospective observational cohort study of patients with OPSCC noted TTMV-HPV DNA to have a sensitivity of 91.5% and a specificity of 100% at the time of diagnosis [39]. A prospective biomarker clinical trial of non-metastatic HPV-related OPSCC noted a negative predictive value of 100%, while two consecutively positive tests had a PPV of 100% [40]. Further prospective and randomized controlled clinical trials will need to be completed before TTMV-HPV DNA becomes routine as a diagnostic or surveillance tool although the data thus far are promising.

## 3. Refractory, Recurrent, and Metastatic HNSCC

### 3.1. Platinum Doublets Plus a Biologic

For patients with unresectable or metastatic HNSCC with no option for surgery or radiation therapy, pembrolizumab with or without chemotherapy is the preferred first-line therapy. This recommendation comes from Keynote-048, a phase III study of patients with incurable HNSCC who were randomized to pembrolizumab alone, pembrolizumab plus chemotherapy (platinum with 5-FU), or the then-standard-of-care EXTREME regimen (cetuximab plus platinum and 5-FU) [13]. Both pembrolizumab-containing arms were compared with the standard-of-care cetuximab-containing arm, but were not directly compared with each other. Pembrolizumab alone resulted in improved OS in patients with a PD-L1 combined positive score (CPS) ≥ 20 (median 14.9 vs. 10.7 months) and a CPS ≥ 1 (median 12.3 vs. 10.3 months), and it was non-inferior with reduced toxicity compared with the standard of care in the total population. Similarly, pembrolizumab plus chemotherapy showed improved OS in patients with a CPS ≥ 20 (median 14.7 vs. 11 months), a CPS ≥ 1 (median 13.6 vs. 10.4 months), and the total population (13.0 vs. 10.7 months). The response rates were 17% for pembrolizumab alone, 36% for pembrolizumab with chemotherapy, and 36% for cetuximab with chemotherapy in the total population [41]. Although clearly an important aspect of care delivery, quality of life was not reported in these trials. This analysis resulted in a dual standard for first-line therapy in patients with CPS-positive tumors; clinically, pembrolizumab alone may be favored for patients with a lower disease burden, fewer disease-related symptoms, and/or reduced functional status, while pembrolizumab with chemotherapy is preferred for those who have higher-volume disease, a greater symptom burden, and/or better fitness.

If immunotherapy is contraindicated, the EXTREME regimen remains a reasonable option for patients who can tolerate triplet therapy, particularly with HPV-negative disease. This combination of cetuximab, platinum, and 5-FU was originally evaluated against platinum doublet chemotherapy in patients with recurrent or metastatic HNSCC [12]. The median OS was significantly prolonged from 7.4 months in the chemotherapy doublet group to 10.1 months in the chemotherapy plus cetuximab group (HR 0.80). The median PFS also improved from 3.3 to 5.6 months. Common adverse events from cetuximab plus chemotherapy included anemia (13%), neutropenia (22%), thrombocytopenia (11%), grade 3 skin reactions (9%), and grade 3–4 infusion-related reactions (3%).

Clinical trials for relapsed/metastatic (R/M) HNSCC have historically used a platinum (cisplatin or carboplatin) chemotherapy backbone plus 5-FU. However, the latter is associated with significant toxicities, including diarrhea and mucositis. The delivery logistics, including the need for central venous access and continuous infusions requiring multiple clinic visits, can also be challenging. In clinical practice, taxanes may be substituted for 5-FU due to convenient administration, potentially better tolerability, and existing data for taxane-based combination regimens in other cancer types. A recent retrospective review demonstrated that the use of taxanes in combination with platinum plus pembrolizumab for metastatic HNSCC has increased over time in the U.S., with higher utilization in academic centers compared with community practices [42]. The authors found no difference in overall survival between patients receiving a taxane versus 5-FU, suggesting that this is a reasonable alternative approach.

### 3.2. Biologic Monotherapy

In patients with R/M HNSCC who are not candidates for chemotherapy or who have disease resistant to chemotherapy, biologic monotherapy can be considered. As above, pembrolizumab monotherapy was approved in the first-line setting based on data from Keynote-048. Several additional studies confirmed durable long-term pembrolizumab responses, even in heavily pretreated patients [43,44,45,46,47]. Nivolumab monotherapy demonstrated similar benefits in patients with recurrent HNSCC, with improved OS compared with single-agent systemic therapy (7.5 vs. 5.1 months; HR 0.70), in Checkmate 141 [14]. Conversely, combination immunotherapy with ipilimumab and nivolumab has not been proven to be effective, as both Checkmate 651 and Checkmate 714 failed to reach their primary end points [48,49]. Similarly, durvalumab, either alone or with tremelimumab, did not yield a significant OS benefit [50,51].

Cetuximab monotherapy is approved for patients who have progressed on immunotherapy or who are ineligible for immune checkpoint inhibition. The initial data supporting this indication came from a 2007 study of patients with R/M HNSCC who progressed to platinum therapy [52]. Patients received weekly cetuximab as a single agent for at least six weeks, after which they were eligible for add-on chemotherapy if their disease progressed. The clinical response was quite modest, with a response rate of 13%, disease control rate of 46%, and median time to progression of 70 days. There were no objective responses in patients who had chemotherapy added on at the later timepoint. A follow-up study in a similar patient population evaluating every-two-week cetuximab dosing found similar results, with a response rate of 11% and a median PFS of 2.2 months [53]. As first-line therapy has evolved to include immune checkpoint inhibition, a re-assessment of cetuximab monotherapy in the second-line setting for patients with immunotherapy-refractory disease is ongoing. Early results suggest that cetuximab may be more effective in patients with prior immunotherapy exposure, with an objective response rate (ORR) of 19% and a median PFS of 3.7 months [54]. This may be due to a delayed response to immunotherapy or synergy between lingering immunotherapy and cetuximab. Additional studies are ongoing to confirm and elaborate on the response to cetuximab given with, or immediately following, anti-PD1 therapy.

### 3.3. Single-Agent and Doublet Chemotherapies

A variety of chemotherapy options exist for patients seeking second-line or later treatment, depending on treatment-resistance patterns, co-morbidities, and functional status. These include platinum plus 5-FU, platinum plus taxane, platinum plus cetuximab, platinum alone, taxane alone, 5-FU alone, and capecitabine alone. The reported response rates are low, typically in the 10–15% range, depending on whether a single-agent or combination therapy is used. Patients considering these options should be evaluated for clinical trials when available.

## 4. Experimental Therapies

While biologics added to chemotherapy have substantially improved outcomes for patients with advanced HNSCC, the response rates and survival gains have been modest. As mentioned above, there are few options available for patients who have progressed to ICI. There is a strong need for effective therapies to improve immunotherapy responses, reverse immunotherapy resistance, and/or treat disease in the immunotherapy-resistant setting. A wide variety of agents are currently under investigation in HNSCC, with a focus on novel and combination immunotherapies (Table 2) and also including multikinase inhibitors, bispecific antibodies, and antibody–drug conjugates (Table 3).

### 4.1. Novel Immunotherapy Combinations 

One approach showing early success in improving the immunotherapy response in HNSCC is to combine ICI with a second immunomodulating agent. One example is eftilagimod alpha, a soluble LAG-3 protein that can act as a major histocompatibility complex (MHC) II agonist and trigger the immune activation of antigen-presenting cells (APCs) and CD8+ T cells. The TACTI-002 trial evaluated eftilagimod alpha plus pembrolizumab in patients with metastatic HNSCC regardless of PD-L1 expression who had disease progression following first-line platinum-based therapy. Of the 39 patients, 30% had an objective response, with a PFS of 2.1 months and a median OS of 8.7 months [55]. The data suggested a reasonable safety profile, and a follow-up randomized study is actively recruiting (NCT04811027). Another target of interest is TGFβ, as the dysregulation of the TGFβ pathway is thought to be an escape mechanism for PD-1/L1-associated therapies [56,57]. Bintrafusp alfa is a bifunctional fusion protein targeting TGFβR2 fused to an IgG monoclonal antibody against PD-L1 that has garnered preclinical attention, but is yet to demonstrate clinical utility. NCT02517398 noted a reasonable safety profile and a 3-year OS of 24% in an expansion cohort of patients with R/M HNSCC who had progressed after platinum therapy [58]. 

Additional combinations are theoretically promising but still undergoing clinical evaluations (Table 1). Unfortunately, not all immunotherapy combinations have borne fruit. GSK609, an agonist of the ICOS immunoglobulin receptor, which provides a co-stimulatory signal for T-cell proliferation and activity, showed early signs of benefit in combination with pembrolizumab in an HNSCC expansion cohort in INDUCE-1 [59]. However, INDUCE-2 found little clinical efficacy, and studies involving GSK609 have subsequently halted [60,61]. Similarly, monalizumab, a novel immune checkpoint inhibitor targeting NKG2a, did not improve the response rate or OS when given with durvalumab plus cetuximab in R/M HNSCC, putting a halt to its further development in this disease [62]. 

### 4.2. TLR Agonists 

Toll-like receptors (TLRs) are essential aspects of the immune activation process, and TLR agonists have been investigated as a potential treatment option for HNSCC. One phase II study using an intralesional TLR9 agonist in combination with pembrolizumab seemed to show some degree of benefit, with responses noted in both injected and non-injected lesions (32% and 29%, respectively) [63]. However, the TLR9 agonist EMD 120108 in combination with cetuximab in the second-line treatment of patients with R/M HNSCC failed to demonstrate efficacy when compared with cetuximab monotherapy [64]. Motolimod, a TLR8 agonist, was given with the EXTREME regimen for R/M HNSCC (Active8) but also did not yield improvements in PFS or OS [65]. A meta-analysis of these three phase II trials, along with data from three additional phase Ib trials, showed an overall lack of a clear benefit for TLR agonists in HNSCC [66].

### 4.3. Tumor-Infiltrating Lymphocytes

T cells are an essential aspect of the immune–oncologic defense against malignancy. Tumor-infiltrating lymphocytes (TILs) are T cells typically isolated directly from primary tumors, then modified or expanded based on certain oncologic targets. In HNSCC, TILs have primarily been studied as prognostication tools and predictors of response to immunotherapy, with some data suggesting their use as a selection tool to determine patients who may best respond to immunotherapy [67]. One study, which evaluated the prognostic use of TILs in oropharyngeal SCC, noted that TILs were associated with disease-specific survival, with a hazard ratio of 2.13 (95% CI 1.14–3.96; *p*  =  0.017), and maintained a prognostic value in both HPV-positive and -negative cohorts [68]. To date, there are few studies evaluating TILs as a treatment. One phase II study evaluating TILs followed by IL-2 for the treatment of R/M disease has been completed, but the results are not yet available (NCT03083873) [69]. Another phase II study evaluating TILs in patients with multiple solid tumors, including HNSCC, is actively recruiting (NCT03645928).

### 4.4. CAR-T

Chimeric antigen receptor T (CAR-T) cell therapy involves genetically modifying T cells to recognize tumor cells in an MHC-independent manner. CAR-T cell therapy has become a staple in the treatment of hematologic malignancies although its translation for use in solid tumors has not been straightforward due to a lack of obvious targets and challenges localizing CAR-T cells into the solid-tumor microenvironment. Side-effect profiles remain a significant barrier in CAR-T therapy as well [70,71]. There are sparse clinical trial data for CAR-T cell therapies in HNSCC. A phase 1 dose-escalation trial evaluated the safety profile and efficacy of CAR-T cells targeting ErbB in HNSCC patients. Cell manufacturing was successful in all 13 cases despite profound lymphopenia, and the authors noted an overall disease control rate of 69%. A limited number of active clinical trials are studying the use of CAR-T cells in HNSCC, with targets including CSPG4 (NCT06096038), Mucin1 (NCT05239143), HER2 (NCT03740256), HPV E7 (NCT05686226), and IL13Ralpha2 (NCT04119024).

### 4.5. Targeted Therapies

HNSCC infrequently harbors targetable genetic findings, but there are drugs in development for those with rare driver alterations. Tipifarnib, a farnesyltransferase inhibitor that disrupts the HRAS pathway, is currently being studied in a phase II trial in patients with R/M HNSCC with a high mHRAS-variant allele frequency. Of 20 evaluable patients, the ORR was 55%, with a median PFS and OS of 5.6 months and 15.4 months, respectively [72]. Another example is bimiralisib, a dual inhibitor of PI3K/mTOR, which showed preclinical activity in NOTCH1-mutant HNSCC cells. Bimiralisib was studied in a small phase II trial of patients with NOTCH1-mutated HNSCC who progressed to first-line platinum-based chemotherapy and immunotherapy (NCT03740100). In this small study, the ORR was 17%, with a median PFS of 5 months and median OS of 7 months [73].

Other targeted therapies have been assessed in HNSCC without the use of biomarker selections. Xevinapant is a potent oral inhibitor of apoptosis that has demonstrated early efficacy in the locally advanced setting when combined with platinum-based chemoradiation. In a phase II study of patients with unresected HNSCC, xevinapant with concurrent chemoradiation produced a 5-year OS rate of 53% compared with 28% in the placebo control group [74]. The xevinapant group was also favored in the 3-year PFS endpoint. A follow-up phase III study, TrilynX, of xevinapant with concurrent standard-of-care chemoradiation is underway for unresected locally advanced HNSCC [75]. In the recurrent/metastatic setting, buparlasib, a highly selective oral PI3K inhibitor, was studied in combination with paclitaxel in patients with HNSCC who had received prior platinum-based therapy (BERIL-1). This trial demonstrated a benefit in the median PFS (4.6 vs. 3.5 months; HR 0.65) and median OS (10.4 vs. 6.5 months; HR 0.72), resulting in a subsequent phase III study (BURAN) to evaluate the combination of paclitaxel with buparlisib compared with paclitaxel alone [76].

### 4.6. Multikinase Inhibitors

Multikinase inhibitors are thought to work through the blockade of multiple potentially overlapping kinase-dependent signaling pathways to prevent tumor progression [77,78]. Targeting VEGF receptors, for example, has demonstrated clinical utility by inhibiting tumor angiogenesis. Lenvatinib is a multikinase inhibitor that inhibits VEGF receptors 1-3, FGFR 1-4, PDGFRa, RET, and c-KIT. KEYNOTE-146 showed a tolerable safety profile and promising antitumor activity with the combination of lenvatinib plus pembrolizumab in advanced solid tumors, reporting an ORR of 72.7% in treatment-naïve patients, 41.2% in previously treated but ICI-naïve patients, and 55.8% in ICI-exposed patients [79]. LEAP-010 explored this combination in R/M HNSCC, and an interim analysis found improved ORR and PFS without OS benefit when compared with pembrolizumab monotherapy [80]. A follow-up study (LEAP-009) will compare the combination of lenvatinib/pembrolizumab against standard-of-care chemotherapy as a second-line treatment (NCT04428151). A similar study of lenvatinib/pembrolizumab adjunctively following definitive chemoradiation in locally advanced HNSCC is also ongoing (NCT05433116). Cabozantinib, a multikinase inhibitor of c-Met, VEGFR2, AXL, and RET, was also shown to be active in combination with pembrolizumab in the recurrent/metastatic setting, with objective responses in 52% of patients and stable disease in another 39% [81]. The COSMIC 021 phase I trial studying cabozantinib in combination with cetuximab and atezolizumab is in progress (NCT03170960), as is the STELLAR-305 phase III study investigating the multikinase inhibitor zanzalintinib with or without pembrolizumab in R/M HNSCC (NCT06082167) [82].

### 4.7. Bispecific Antibodies

Multiple bispecific antibodies are under investigation in HNSCC with the aim of co-localizing complementary therapies within the tumor microenvironment. BCA101 is a first-in-class bifunctional EGFR antibody with a TGFβ immune-modulating payload. The 731MO trial demonstrated the tolerable safety profile and early clinical efficacy of BCA101 as mono- and combination therapy with pembrolizumab in advanced solid tumors. In an expansion cohort, patients with R/M HNSCC who had not received prior therapy were observed to have an ORR of 44% and a clinical benefit rate of 67%, with 58% of HPV-negative patients achieving a response [83]. This regimen is actively recruiting for a phase I/Ib study, NCT04429542. Similarly, petosemtamab, a bispecific antibody targeting EGFR and LGR5, demonstrated an ORR of 37.2% and a disease control rate of 72.1% in the HNSCC cohort of the phase 1/2 MCLA-158-CL01 trial (NCT03526835) [84]. These preliminary data led to an FDA breakthrough therapy designation and a follow-up phase II study in combination with pembrolizumab as first-line therapy in patients with recurrent/metastatic disease. Finally, volrustomig is a monovalent bispecific antibody engineered against PD-1 and CTLA-4, with an increased CTLA-4 blockade on PD-1-positive activated T cells. A phase 1/2 study demonstrated clinical efficacy and overall tolerability in the treatment of RCC and NSCLC (NCT03530397), and a follow-up phase III study, eVOLVE-HNSCC, is assessing volrustomig as an adjuvant therapy after chemoradiation for locoregional disease and is actively recruiting [85]. 

### 4.8. Therapeutic Vaccines

As a substantial portion of oropharyngeal HNSCC is caused by HPV, there is great interest in HPV E6- and/or E7-expressing therapeutic vaccines for the management of advanced HPV-positive malignancies, including HNSCC. Clinical studies remain limited, but thus far have generated mixed results. AXAL was found to cause prohibitively high rates of adverse events, resulting in the early termination of multiple studies. BNT113 is actively under investigation in combination with pembrolizumab (NCT04534205). ISA101, a synthetic long-peptide vaccine derived from E6 and E7 proteins, demonstrated apparent OS benefits, including durable responses, when given with nivolumab in HPV16-positive HNSCC [86,87]. Unfortunately, similar studies of ISA101b in combination with cemiplimab (anti-PD-1) showed less-clear benefits [88,89]. Additional trials are ongoing to evaluate ISA101b with pembrolizumab/cisplatin-based therapy (NCT04369937) and in combination with cemiplimab in HPV-positive oropharyngeal HNSCC (NCT03669718). MEDI0457, a vaccine containing DNA plasmids encoding E6, E7, and the immune activator IL-12, also produced encouraging results in combination with durvalumab, with an ORR of 27.6%, a median PFS of 3.5 months, and a median OS of 29.2 months (NCT03162224) [90]. Lastly, PDS0101 in combination with pembrolizumab suggested a promising clinical benefit, with a median PFS of 10.4 months and a 12-month OS of 87.1% [91]. PDS0101 is under further evaluation as a triple combination with M9241 (IL-12 immunocytokine) and bintrafusp alfa (NCT04287868). These ongoing studies will be crucial in determining what role therapeutic vaccines will play in the management of HPV-positive HNSCC.

## 5. Antibody–Drug Conjugates

Antibody–drug conjugates (ADCs) are a class of drugs designed to deliver a cytotoxic chemotherapy payload to specific targets using antibodies, thereby limiting the toxicity of conventional systemic chemotherapy. Since 2009, several ADCs have been approved by the FDA for the treatment of other solid-tumor subtypes, but there are none so far for HNSCC [92]. Several ADCs are being investigated in HNSCC (Table 2).

### 5.1. Bivatuzumab Mertansine

Bivatuzumab mertansine was an early-generation ADC, delivering mertansine to CD44-positive cells. A phase I study was terminated early due to severe dermatologic adverse events. Despite this, the trial suggested early efficacy data, with several patients demonstrating a PR [93]. There are no active clinical trials evaluating bivatuzumab in HNSCC at this time.

### 5.2. Enfortumab Vedotin

Enfortumab vedotin (EV) is a nectin-4-targeted ADC with an MMAE payload hypothesized to have clinical activity in HNSCC given HNSCC’s elevated expression of nectin-4. EV-202 is a multicohort open-label phase 2 study evaluating EV in numerous previously treated locally advanced or metastatic solid tumors, including an HNSCC cohort. In the HNSCC cohort, patients all had prior disease progression after 1 platinum-based therapy and anti-PD-1/PD-L1 therapy. The authors observed an ORR of 23.9%, with a median duration of response not reached. The median PFS was 3.94 months and the median OS was 5.98 months, with notable dermatologic reactions and peripheral neuropathy side effects. This study highlights the potential use of EV monotherapy in previously treated advanced HNSCC [94,95]. EV-202 (NCT04225117) is actively recruiting.

### 5.3. Sacituzumab Govitecan

Sacituzumab govitecan (SG) is a Trop-2-directed ADC linked to a topoisomerase inhibitor. TROPiCS-03 is an open-label multicohort phase 2 basket study evaluating the use of SG monotherapy in locally advanced and metastatic solid tumors, which includes an HNSCC cohort. Patients in the HNSCC cohort had disease progression after prior platinum-based chemotherapy and anti-PD-L1 therapy, and received SG. The results demonstrated an ORR of 16% and a combined benefit rate of 26%, with a median duration of response of 4.2 months and a median PFS of 4.1 months [96,97]. There appears to be some clinical activity with SG although further study remains.

### 5.4. SGN-B6A

HNSCC is among several solid-tumor subtypes that have demonstrated a high ITGB6 expression [98]. SGN-B6A is an ADC directed against ITGB6 with an MMAE payload. In a phase I study evaluating a mixed-patient population with non-small-cell lung cancer, HNSCC, and esophageal cancer, SGN-B6A demonstrated a reasonable safety profile and some preliminary antitumor activity, with the HNSCC expansion cohort demonstrating an ORR of 31.6% [99]. Expansion cohorts are ongoing and recruiting in an active clinical trial (NCT04389632).

### 5.5. Tisotumab Vedotin

Tisotumab vedotin (TV) is an ADC-targeting tissue factor with an MMAE payload. InnovaTV-207 is a global phase 2 multicohort study evaluating TV as monotherapy, with Part C evaluating its use in patients with R/M HNSCC. Early data show an ORR of 32.5% and a median duration of response of 5.6 months. TV appears to have antitumor activity in pretreated patients, with a reasonable safety profile [100]. 

## 6. Conclusions and Future Directions

Medical therapy for head and neck squamous cell carcinoma has significantly changed with the incorporation of immunotherapy and targeted therapies. For locoregional disease, treatment is primarily focused on surgical resection and radiation, with consideration for platinum-based chemotherapy. The benefits of the addition of immunotherapy in the locoregional space remain unclear. For patients with R/M HNSCC, the data for immunotherapy are clearer; the addition of pembrolizumab, either as monotherapy or in combination with chemotherapy, has a demonstrable clinical benefit and should be considered as first-line. Cetuximab can also be considered for appropriate patients. Investigational therapies in HNSCC have produced mixed results to date, and it remains to be seen whether there is clinical utility for the use of many of the therapies discussed in this review. Any consideration for treatment with novel therapies should be individualized, with consideration for cost-effectiveness and accessibility. Significantly, further research remains to be conducted to determine whether there is a role for many of these experimental therapies in HNSCC.

## Figures and Tables

**Table 1:** Brief review of clinical outcomes of significant systemic therapy trials in HNSCC.

Reference	Study Name	Study Population	Intervention Groups	Primary Outcome(s)
***Locoregional Disease***				
Adelstein, et al., 2003 [4]	Head and Neck Intergroup Phase III Study	Unresectable HNSCC	A: RT alone	Three-year projected OS: A: 23% B: 37% C: 27%
			B: RT + concurrent bolus cisplatin	
			C: Split-course RT + concurrent 5-FU + bolus cisplatin	
Bourhis, et al., 2012 [11]	GORTEC 99-02	Locally advanced (stage III and IV non-metastatic) HNSCC	7 weeks RT + 3 cycles carboplatin + 5-FU	Accelerated chemoRT has no PFS benefit Conventional chemoRT improves PFS vs. very accelerated RT (HR 0.82)
			6 weeks RT + 2 cycles carbo/5-FU	
			RT alone for 3.5 weeks	
***Refractory, Recurrent, and Metastatic Disease***				
Vermorken, et al., 2008 [12]	EXTREME	Untreated R/M HNSCC	Platinum + 5-FU + cetuximab	Median OS 10.1 vs. 7.4 Median PFS 5.6 vs. 3.3 RR 36% vs. 20%
			Platinum + 5-FU (control)	
Burtness, et al., 2019 [13]	KEYNOTE-048	Untreated incurable R/M HNSCC	Pembrolizumab alone	CPS ≥ 20: Pem alone: median OS 14.9 vs. 10.7 Pem + chemo: 14.7 vs. 11 CPS ≥ 1: Pem alone: 12.3 vs. 10.3 Pem + chemo: 13.6 vs. 10.4
			Pembrolizumab + platinum + 5-FU	
			Cetuximab + platinum + 5-FU (control)	
Ferris, et al., 2016 [14]	Checkmate 141	Recurrent HNSCC, with progression within 6 months of platinum-based chemotherapy	Nivolumab	Median OS 7.5 vs. 5.1 Median PFS 2.0 vs. 2.3 RR 13.3% vs. 5.8%
			Standard single-agent therapy (control)	

OS and PFS are presented in months and reported as intervention vs. control. Abbreviations: R/M: recurrent/metastatic; RT: radiation therapy; 5-FU: 5-Fluorouracil; Pem: pembrolizumab; OS: Overall survival; PFS: progression-free survival; HR: hazard ratio; RR: response rate; CPS: combined positive score; chemoRT: chemoradiotherapy.

**Table 2:** Active clinical trials investigating immunotherapy-based treatments in HNSCC.

Trial ID	Phase	Investigational Therapies	Study Population	Status
NCT04754321	I	Pembrolizumab + Salvage Surgery + RT	Recurrent or Persistent	Recruiting
NCT05222932	I	TILT-123 and Avelumab	R/M	Recruiting
NCT05635643	I	CHS-114	R/M	Recruiting
NCT02988960	I	ABBV-927 +/− ABBV-181	LA; R/M	Not Recruiting
NCT04999202	I	BAY2416964 + Pembrolizumab	R/M	Not Recruiting
NCT03283605	I/II	Durvalumab + Tremelimumab + SBRT	Metastatic	Not Recruiting
NCT03317327	I/II	Nivolumab + RT	Recurrent or 2nd Primary	Recruiting
NCT04555837	I/II	Alisertib + Pembrolizumab	R/M; HPV+	Not Recruiting
NCT06319963	I/II	Lenti-HPV-07	R/M	Not Yet Recruiting
NCT04815720	I/II	Pepinemab + Pembrolizumab	LA; R/M	Recruiting
NCT04977453	I/II	GI-101	Metastatic	Recruiting
NCT05597839	I/II	DF9001	R/M	Recruiting
NCT04198766	I/II	INBRX-106 +/− Pembrolizumab	LA; Metastatic	Recruiting
NCT05086692	I/II	MDNA11 + Pembrolizumab	LA; Metastatic	Recruiting
NCT05592626	I/II	STAR0602	LA; R/M	Recruiting
NCT06170697	II	Camrelizumab + CT + RT	R/M; Short-Term Post-Op Progression	Recruiting
NCT03546582	II	RT +/− Pembrolizumab	Recurrent or 2nd Primary	Recruiting
NCT04326257	II	Nivolumab + (Relatlimab or Ipillimumab)	R/M	Not Recruiting
NCT03341936	II	Nivolumab + Lirilumab + Salvage Surgery	Recurrent	Not Recruiting
NCT06239220	II	PD-L1 t-haNK + N-803 + Cetuximab	LA; R/M	Recruiting
NCT04428151	II	Lenvatinib + Pembrolizumab	R/M	Recruiting
NCT06062420	II	Dostarlimab +/− Other Immunotherapies	R/M	Recruiting
NCT03993353	II	Pembrolizumab + Tadalafil	R/M	Recruiting
NCT04260126	II	PDS0101 + Pembrolizumab	R/M; HPV+	Not Recruiting
NCT06052839	II	Pembrolizumab + CT	R/M	Recruiting
NCT05260671	II	Penpulimab + Cetuximab	R/M	Recruiting
NCT03946358	II	Atezolizumab and UCPVax	LA; Metastatic; HPV+	Not Recruiting
NCT03228667	II	N-803 + (Pembrolizumab or Nivolumab)	R/M	Not Recruiting
NCT05686226	II	E7 TCR-T cells	R/M; HPV+	Recruiting
NCT04802876	II	Spartalizumab or Tislelizumab	R/M	Recruiting
NCT04357873	II	Pembrolizumab + Vorinostat	R/M	Not Recruiting
NCT06295731	II/III	Pembrolizumab +/− INBRX-106	R/M	Recruiting
NCT06513884	II/III	HB-202/HB-201 + Pembrolizumab	R/M Oral SCC; HPV+	Not Yet Recruiting

Abbreviations: R/M: recurrent/metastatic; LA: locally advanced; RT: radiation therapy; SBRT: stereotactic body radiation therapy; HPV: human papillomavirus.

**Table 3:** Active clinical trials investigating antibody–drug conjugates in HNSCC.

Trial ID	Phase	Investigational Therapies	Antibody Target	Drug Conjugate	Study Population	Status
NCT06549816	I	Sigvotatug vedotin	Integrin β-6	MMAE	Metastatic/unresectable advanced solid tumors	Not yet recruiting
NCT06147037	I	[225Ac]-FPI-2068	EGFR & cMET	Actinium-225	R/M solid tumors, including HNSCC	Recruiting
NCT04152499	I/II	Sacituzumab tirumotecan (SKB264)	TROP2	Belotecan	Locally advanced unresectable/metastatic solid tumors, including HNSCC	Recruiting
NCT06465069	Ia/Ib	LY4052031	Nectin-4	Novel TOPO 1 inhibitor	Advanced/metastatic solid tumors, including HNSCC	Recruiting
NCT06238479	Ia/Ib	LY4101174	Nectin-4	MMAE	Advanced/recurrent/metastatic solid tumors, including HNSCC	Recruiting
NCT06509997	II	MRG003 + dalpicicilip	EGFR	MMAE	CDKN2A-variant R/M HNSCC	Not yet recruiting
NCT05271604	II	Ozuriftamab vedotin (BA3021)	ROR2	MMAE	ROR-2-expressing R/M HNSCC with prior PD-1/L1 failure	Recruiting
NCT06530914	II	MRG003 + pucotenlimab +/− cisplatin	EGFR	MMAE	EGFR-positive locally advanced HNSCC; neoadjuvant	Not yet recruiting
NCT05751512	II	MRG003 vs. cetuximab/methotrexate	EGFR	MMAE	R/M HNSCC; 2nd or 3rd line therapy; prior failed PD-1/L1	Not yet recruiting
NCT06003231	II	Disitamab vedotin	HER2	MMAE	HER2 IHC expression >1 + advanced/metastatic solid tumors, including R/M HNSCC	Recruiting

Abbreviations: MMAE: monomethyl auristatin.
